# Standardized Papillary Hemospray Application for Immediate Post-Endoscopic Sphincterotomy Bleeding: A Historical-Control Cohort Study (with Video)

**DOI:** 10.1055/a-2936-2343

**Published:** 2026-08-25

**Authors:** Takahito Sagawa, Takeshi Ogura, Saori Ueno, Nobu Nishioka, Junichi Nakamura, Nobuhiro Hattori, Kimi Bessho, Takafumi Kanadani, Masahiro Yamamura, Nga Nguyen Trong, Kim Chhun Ly, Ahmad F. Aboelezz, Yi-Jun Liao, Szu-Chia Liao, Hiroki Nishikawa

**Affiliations:** 1 Pancreatobiliary Advanced Medical Center 13010 Osaka Medical and Pharmaceutical UniversityTakatsukiOsaka PrefectureJapan; 2 2nd Department of Internal Medicine 13010 Osaka Medical and Pharmaceutical UniversityTakatsukiOsaka PrefectureJapan; 3 Endoscopic DepartmentTrong Nam Cancer HospitalHanoi65Viet Nam; 4 Department of Gastroenterology 540452 Calmette HospitalPhnom PenhPhnom PenhCambodia; 5 Department of Internal Medicine, Gastroenterology and Hepatology Unit 68781 Tanta UniversityTantaEgypt; 6 Division of Gastroenterology and HepatologyDepartment of Internal Medicine 40293 Taichung Veterans General HospitalTaichungTaichung CityTaiwan

**Keywords:** pancreatobiliary (ERCP/PTCD), ERC topics, strictures

## Abstract

**Background and aim** To evaluate the feasibility and short-term safety of Hemospray as first-line hemostatic therapy for immediate post-endoscopic sphincterotomy (EST) bleeding using a standardized papillary application protocol, and to compare outcomes with a historical balloon-tamponade group.

**Methods** The primary outcome was initial endoscopic hemostasis after first-line therapy. Key secondary outcomes were 30-day rebleeding, overall endoscopic hemostasis, rescue-device use, adverse events within 7 days, and exploratory device-cost comparison. Primary analyses included all eligible patients, categorized according to the first-line hemostatic therapy actually received, irrespective of subsequent rescue therapy.

**Results** Among 1666 EST procedures, 66 bleeding episodes required endoscopic hemostasis and were included (Hemospray, 25/498 [5.0%]; balloon tamponade, 41/1168 [3.5%]); 21 episodes of self-limited oozing were excluded. Initial hemostasis was achieved more often with Hemospray than with balloon tamponade (96.0% vs. 73.2%; risk difference 22.8%, 95% CI 4.3–41.4; *P* = 0.023). Firth-penalized multivariable analysis supported this direction (adjusted odds ratio 7.7, 95% CI 1.5–84.2; *P* = 0.013), although precision was limited by quasi-separation. In the propensity-score–matched analysis (20 pairs), initial hemostasis remained numerically higher with Hemospray (95.0% vs. 80.0%; *P* = 0.375). Overall endoscopic hemostasis was achieved in all patients. All rebleeding occurred within 24 h and was numerically more frequent with Hemospray (12.0% vs. 2.4%; *P* = 0.14), with no additional events through 30 days. Adverse events occurred in 12.0% vs. 22.0% ( *P* = 0.36).

**Conclusions** A standardized Hemospray-first protocol was associated with improved initial bleeding control and reduced rescue-device use in persistent nonpulsatile post-EST oozing. However, overall endoscopic hemostasis was identical between groups, and hemostatic durability remains uncertain.

## Introduction

Endoscopic retrograde cholangiopancreatography (ERCP) is the reference-standard modality for the diagnosis and treatment of pancreatobiliary diseases. 1 Endoscopic sphincterotomy (EST) is routinely performed before bile duct stone removal, self-expandable metal stent (SEMS) deployment, or insertion of a cholangioscope. Immediate post-EST bleeding is a well-recognized adverse event, with a reported incidence of 1–12.1%. 2 3 4

Several techniques have been reported to achieve endoscopic hemostasis, including thermal coagulation, balloon tamponade, SEMS deployment, and hemoclipping. 5 6 7 8 9 More recently, the application of a self-assembling peptide (PuraStat; 3-D Matrix, Ltd, Tokyo, Japan) has been reported as an additional hemostatic option. 10 11 Application of PuraStat to the ampulla of Vater, however, can be technically challenging because of the anatomical configuration of the papilla and the limited working space within the duodenum, although promising results have been reported. 12 13

The endoscopic hemostatic powder TC-325 (Hemospray; Cook Medical, Bloomington, IN, USA) has recently become available as a topical hemostatic agent. Randomized controlled trials indicate that Hemospray is a promising option for achieving immediate hemostasis in patients with active gastrointestinal bleeding. 14 15 TC-325 is an inorganic, nonabsorbable powder that rapidly absorbs water on contact and adheres to the bleeding surface, forming a cohesive barrier that seals the lesion and facilitates clot formation.

However, concerns remain regarding local dilution of clotting factors and dislodgement of formed clots, which may limit long-term hemostatic durability. 16 17 In addition, the safety of applying Hemospray directly to the ampulla of Vater has not been well established; because the powder may spread across the papillary region, theoretical concerns exist regarding obstruction of the biliary or pancreatic ductal orifices.

Application within the duodenum is also technically demanding because of the narrow lumen and the constant presence of bile and pancreatic juice. To address these issues, we developed a standardized technical protocol emphasizing controlled delivery and papillary visualization. The present study aimed to evaluate the feasibility and short-term safety of Hemospray for immediate post-EST bleeding using this standardized protocol, and to compare initial hemostasis, rescue-device use, and exploratory device-cost comparison with those of historical balloon tamponade, using Firth-penalized multivariable regression and propensity-score matching to mitigate temporal and indication bias.

## Patients and Methods

### Study Design and Patients

This single-center, retrospective, historical-control cohort study screened all EST procedures performed at Osaka Medical and Pharmaceutical University Hospital between June 2024 and December 2025. Eligible patients were consecutive patients who underwent endoscopic hemostasis for immediate post-EST bleeding according to the institutional treatment criteria described below. Between June 2024 and August 2025, balloon tamponade was used as the first-line hemostatic technique, whereas from September 2025 onward, a standardized papillary Hemospray protocol was adopted as the first-line technique. All eligible patients were included in the primary analysis and were analyzed according to the first-line hemostatic modality actually received, irrespective of subsequent rescue therapy. Because this was a retrospective study of the initial clinical experience with the standardized Hemospray protocol, no formal sample-size calculation was performed.

The study was approved by the Human Research Committee of Osaka Medical and Pharmaceutical University (approval no. 2025-218) and conformed to the 2024 revision of the Declaration of Helsinki. The requirement for written informed consent was waived owing to the retrospective design; an opt-out notice was posted on the hospital website.

### Indications for Endoscopic Hemostasis

At our institution, EST was generally performed by endoscopists with 5 years or less of ERCP experience under the direct supervision of an expert pancreatobiliary endoscopist. Hemostatic treatment for immediate post-EST bleeding was performed within the same supervisory framework. The operator eligibility and expert-supervision structure remained unchanged throughout the balloon-tamponade and Hemospray periods.

All bleeding episodes included in this study consisted of nonpulsatile oozing from the sphincterotomy site. Oozing was initially managed with irrigation and suction, followed by endoscopic observation for 3 min. Hemostatic treatment was initiated only when oozing persisted throughout this observation period. Self-limited oozing or visible blood that resolved spontaneously within 3 min without hemostatic intervention was excluded. No pulsatile or projectile bleeding was observed in either study period.

The first-line hemostatic modality differed according to the study period: balloon tamponade was used during the historical-control period, whereas Hemospray was adopted as the standardized first-line modality from September 2025 onward. When first-line treatment failed, rescue therapy was selected according to the persistence of bleeding and the judgment of the treating endoscopist.

### Standardized Papillary Hemospray Protocol



**Video 1** The video demonstrates each step of the standardized papillary Hemospray protocol: (i) priming the catheter by advancing the powder until it reaches the distal tip; (ii) disconnecting the suction line and clearing residual water from the working channel by forceful air flushing; (iii) confirming catheter patency with a low-volume test burst after the catheter emerges into the duodenal lumen; and (iv) delivering Hemospray in a sweeping tangential pattern with the endoscope positioned slightly to the left and superior to the papilla, achieving uniform coverage of the entire papillary surface.


**Fig. 1:**
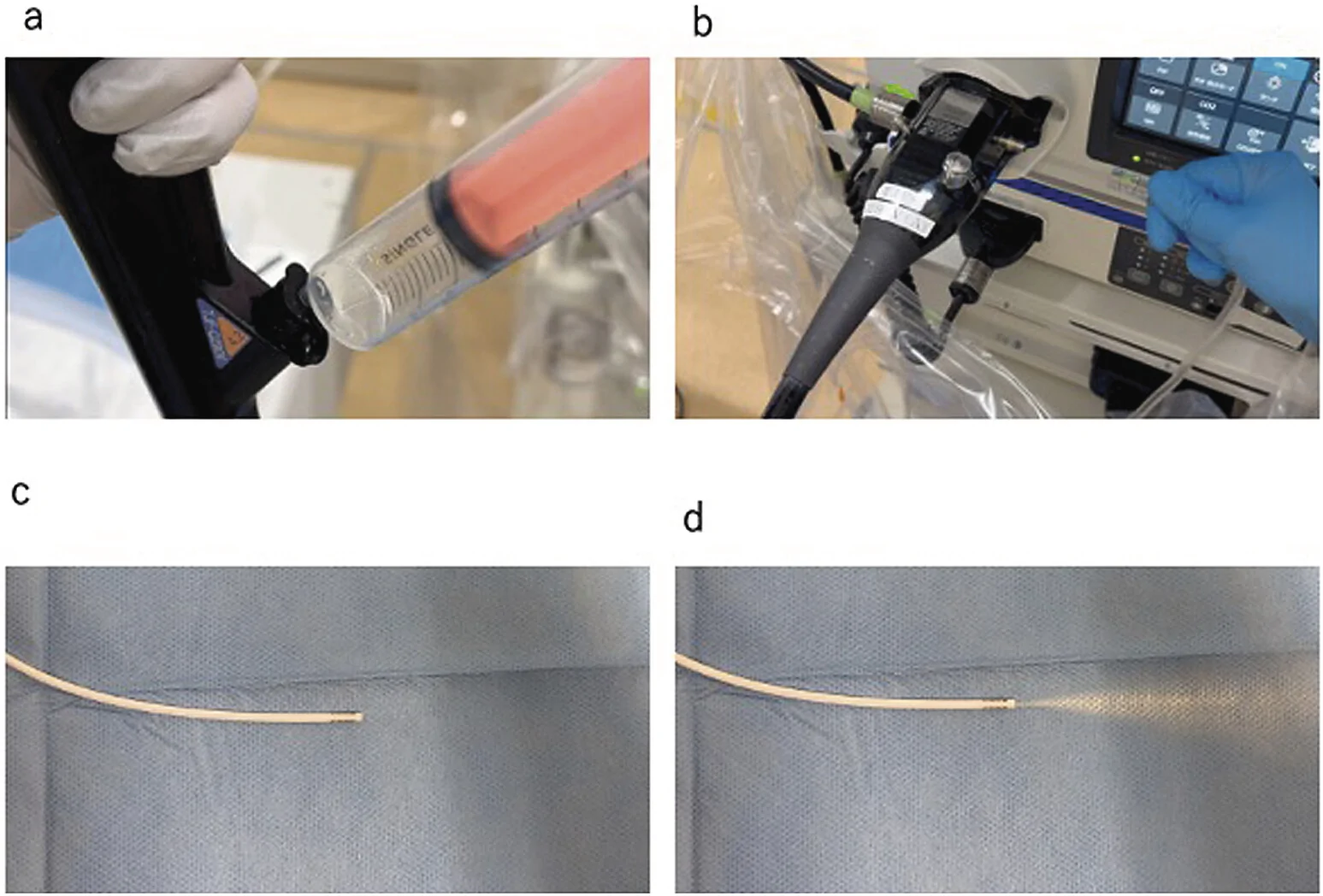
Preparation of Hemospray application. ( **A** ) Residual water is expelled by forcefully flushing air through a syringe connected to the channel port until complete dryness is confirmed. ( **B** ) The suction line is disconnected during preparation to prevent inadvertent aspiration of duodenal fluid that could compromise powder delivery. ( **C** , **D** ) The Hemospray catheter is primed before insertion by advancing the powder until it visibly reaches the distal tip ( **C** , before; **D** , after priming).

**Fig. 2:**
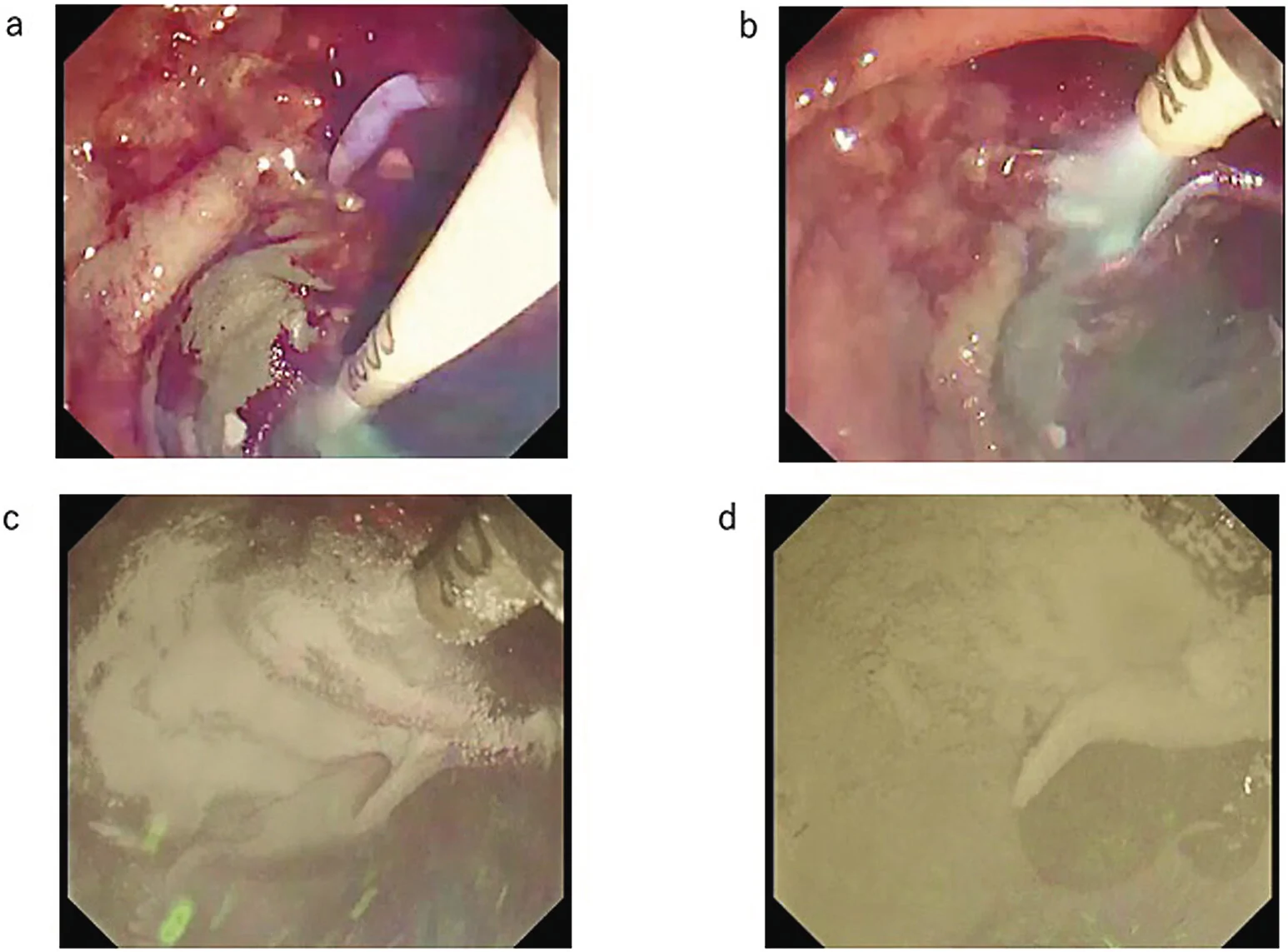
Technical tips for papillary Hemospray application. ( **A** ) After the catheter emerges into the duodenal lumen, a brief low-volume test burst is delivered. ( **B** ) The endoscope is positioned slightly to the left and superior to the papilla, with a gentle downward viewing angle. ( **C** ) The powder is applied in a sweeping manner to achieve uniform coverage of the entire papillary surface. ( **D** ) Excessive focal or prolonged spraying leads to thick powder clumping and should be avoided.

Figs. 1 and 2 illustrate the technical protocol. Because Hemospray aggregates rapidly on contact with moisture and may occlude the catheter, meticulous preparation, and strict moisture control are essential. Before device insertion, residual fluid within the working channel is expelled by forcefully flushing air through a syringe connected to the channel port until complete dryness is confirmed ( Fig. 1A ). Suction is disconnected during this step to prevent inadvertent aspiration of duodenal fluid ( Fig. 1B ). The channel is kept completely dry throughout preparation. The catheter is then primed by advancing the powder until it visibly reaches the distal tip ( Fig. 1C,D ), eliminating any air gap or moisture and minimizing the risk of clogging at initial deployment. Premature activation inside the working channel is carefully avoided.

Once the catheter emerges into the duodenal lumen, a brief, low-volume test burst is delivered ( Fig. 2A ) to confirm patency and to displace any residual moisture at the tip. Full therapeutic application is initiated only after smooth and consistent powder flow is confirmed. For papillary delivery, the duodenoscope is positioned slightly to the left and superior to the papilla, with a gentle downward viewing angle ( Fig. 2B ), allowing controlled tangential delivery rather than direct frontal impact and reducing focal powder accumulation. The powder is then applied in a sweeping manner to achieve uniform coverage of the entire papillary surface ( Fig. 2C ). Excessive pressure or prolonged continuous spraying at a single point is avoided because it results in thick clumping ( Fig. 2D ). These refinements optimize powder dispersion, prevent catheter occlusion, and promote reliable hemostatic coverage of the papillary region ( Video 1 ).

### Outcomes and Definitions

The primary outcome was initial endoscopic hemostasis. According to institutional practice, the sphincterotomy site was observed for approximately 3 min after completion of first-line hemostatic treatment. For this retrospective analysis, initial hemostasis was defined as documented cessation of bleeding after first-line treatment without the need for immediate rescue hemostatic therapy during the index procedure. Failure was defined as persistent or recurrent bleeding requiring immediate additional hemostatic treatment. The exact duration of post-treatment observation was explicitly documented in only a subset of cases. Secondary outcomes were: (i) cumulative rebleeding at 24, 72 h, and 30 days after the index procedure, defined as clinically evident gastrointestinal bleeding requiring endoscopic, radiologic, or surgical intervention or associated with a decrease in hemoglobin of ≥2 g/dL; (ii) overall endoscopic hemostasis, defined as final hemostasis without recourse to surgery or interventional radiology; (iii) use of a rescue hemostatic device; (iv) adverse events within 7 days; and (v) an exploratory device-cost comparison. To assess causality of pancreatobiliary adverse events, computed tomography (CT) was performed in patients with clinical suspicion of such complications. Ductal obstruction possibly attributable to Hemospray was defined as new or worsening dilation of the bile or pancreatic duct compared with preprocedural imaging.

All bleeding episodes included in the analysis consisted of persistent nonpulsatile oozing, defined as continuous nonpulsatile bleeding from the sphincterotomy site that persisted for 3 min despite irrigation, suction, and endoscopic observation. No pulsatile or projectile bleeding was observed. Severity was categorized according to the American Society for Gastrointestinal Endoscopy lexicon 18 : moderate events required transfusion, intensive care unit (ICU) admission, angiographic intervention, or hospitalization of 4–10 days; severe events required surgery, ICU stay longer than 24 h, or hospitalization exceeding 10 nights; all others were mild.

Periprocedural antithrombotic management followed a standardized institutional protocol. Aspirin was continued throughout the periprocedural period in patients at high thrombotic risk and was otherwise interrupted 5–7 days before EST. Thienopyridines were interrupted 5–7 days before EST. Warfarin was withheld for 5 days, and patients at high thromboembolic risk (e.g., mechanical valve, recent venous thromboembolism, CHADS₂ score ≥3) were bridged with unfractionated or low-molecular-weight heparin during the warfarin-free window; heparin was stopped 4–6 h before the procedure. Heparin bridging was not used for DOACs or for antiplatelet agents. DOACs were withheld for 2 days. In patients receiving dual antiplatelet therapy, aspirin was continued while the thienopyridine was interrupted. Antithrombotic therapy was resumed on postoperative day 1 if no bleeding recurred. In this cohort, no patient in either group required heparin bridging during the periprocedural window.

### Exploratory Device-cost Comparison

An exploratory device-cost comparison was performed; this was not a formal cost-effectiveness evaluation. Device cost was defined as the sum of the first-line hemostatic device cost and the cost of any rescue hemostatic devices used during the index procedure. Hospitalization, transfusion, readmission, personnel, procedural, and indirect costs were not included. Mean device costs and the between-group mean difference were calculated, with uncertainty assessed using nonparametric bootstrap resampling with 10,000 iterations.

### Statistical Analysis

The distributions of baseline continuous variables were assessed using the Shapiro–Wilk test. Because several variables were non-normally distributed and the group sizes were relatively small, baseline continuous variables were reported as medians with interquartile ranges and compared using the Mann–Whitney *U* test. Categorical variables were reported as counts and percentages and compared using the χ ^2^ test or Fisher’s exact test, as appropriate. Risk differences and 95% CIs were calculated using the Newcombe hybrid score method. Because the Hemospray group contained only one event of primary-outcome failure, quasi-separation precluded standard maximum-likelihood logistic regression; we therefore used Firth-penalized logistic regression adjusted for prespecified baseline (pretreatment) covariates only: age, antithrombotic therapy (yes/no), EST incision size (small vs. medium/large), EST indication (bile duct stones, biliary drainage, or other), and the Glasgow–Blatchford score. Post-EST bleeding severity (mild vs. moderate) was intentionally not included, because under the ASGE lexicon 18 it is defined partly by post-treatment actions (transfusion, ICU admission, hospitalization length) and is therefore not a baseline confounder. Given quasi-separation, the Firth model was interpreted primarily for the direction of effect rather than for effect-size estimation. To further mitigate temporal and indication bias inherent in historical-control comparisons, we additionally performed 1:1 nearest-neighbor propensity-score matching (caliper 0.2 × SD of the logit of the propensity score, without replacement) using the same covariates; covariate balance was assessed by standardized mean differences. The propensity-score–matched analysis was prespecified as a supportive sensitivity analysis rather than a confirmatory comparison. Because only 20 matched pairs were obtained, the analysis had limited statistical power and was used primarily to assess whether the direction of the association was consistent with the primary analysis.

## Results

### Patient Characteristics

**Fig. 3:**
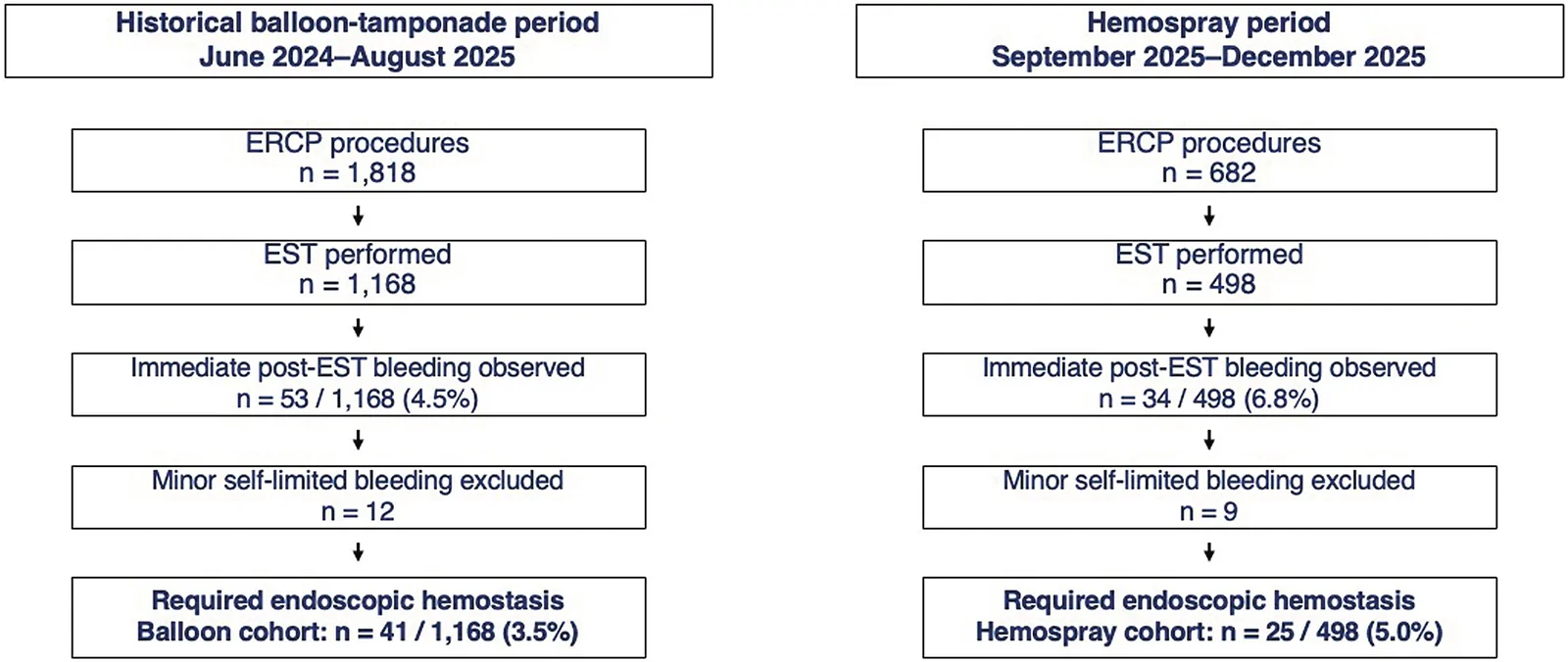
Patient flow according to study period. During the balloon-tamponade period, immediate oozing occurred after 53 of 1168 EST procedures; 12 self-limited episodes were excluded, and 41 patients requiring hemostasis were included. During the Hemospray period, immediate oozing occurred after 34 of 498 EST procedures; 9 self-limited episodes were excluded, and 25 patients requiring hemostasis were included.

During the historical balloon-tamponade period, 1818 ERCP procedures were performed, including 1168 EST procedures. Immediate bleeding occurred after 53 EST procedures (4.5%). Twelve episodes of self-limited oozing resolved within the 3-minute observation period without hemostatic intervention and were excluded. The remaining 41 patients required endoscopic hemostasis, corresponding to an incidence of 3.5% (41/1168). During the Hemospray period, 682 ERCP procedures were performed, including 498 EST procedures. Immediate bleeding occurred after 34 EST procedures (6.8%). Nine episodes of self-limited oozing resolved within the 3-minute observation period and were excluded. The remaining 25 patients required endoscopic hemostasis, corresponding to an incidence of 5.0% (25/498). The incidence of immediate post-EST bleeding requiring hemostasis did not differ significantly between the two periods (3.5% vs. 5.0%; *P* = 0.170). Overall, 66 of 1,666 EST procedures (4.0%) were associated with immediate bleeding requiring endoscopic hemostasis. The higher procedural volume during the Hemospray period followed the establishment of a structured referral pathway and its dissemination to referring institutions. Patient flow is shown in Fig. 3 .

The Hemospray group comprised 25 patients (12 men and 13 women), with a median age of 78 years (IQR, 75–81 years), whereas the balloon-tamponade group comprised 41 patients (24 men and 17 women), with a median age of 76 years (IQR, 65–81 years). Baseline characteristics are shown in Table 1 . The groups were similar with respect to age, sex, ECOG performance status, Charlson Comorbidity Index, antithrombotic therapy, international normalized ratio, platelet count, hemoglobin, and Glasgow–Blatchford score. The median Glasgow–Blatchford score was 1 (IQR, 1–5) in the Hemospray group and 1 (IQR, 0–3) in the balloon-tamponade group ( *P* = 0.28). The distribution of EST incision size did not differ significantly between the groups ( *P* = 0.79). In the Hemospray group, 16 patients (64.0%) underwent small EST and 9 (36.0%) underwent medium EST; no patient underwent large EST. In the balloon-tamponade group, 24 patients (58.5%) underwent small EST, 16 (39.0%) underwent medium EST, and 1 (2.4%) underwent large EST. The balloon-tamponade group contained a greater proportion of biliary drainage cases, whereas the Hemospray group included more bile duct stone removal and ampullary/cholangioscopy procedures. This residual case-mix difference was addressed by the adjusted and propensity-score–matched analyses. Initial hemostasis and rescue device use Clinical outcomes are summarized in Table 2 . Among patients receiving antithrombotic therapy, drug management (interrupted/heparin bridge/uninterrupted) was 4/0/3 in the Hemospray group and 6/0/4 in the balloon group ( *P* = 0.96); no patient in either group required heparin bridging during the periprocedural window. The distribution of EST incision size did not differ between groups (small 64.0% vs. 58.5%; *P* = 0.79). Nearly all cases were classified as mild in severity according to the ASGE lexicon (96.0% vs. 97.6%).

**Table 1:** Baseline characteristics of patients with immediate post-EST bleeding.

Characteristic	Hemospray ( *n* = 25)	Balloon ( *n* = 41)	*P* -value
Age, years, median (IQR)	78 (75–81)	76 (65–81)	0.31
Male sex, *n* (%)	12 (48.0)	24 (58.5)	0.45
ECOG performance status, median (IQR)	1 (0–2)	1 (0–2)	0.70
Charlson Comorbidity Index, median (IQR)	7 (4–9)	6 (4–8)	0.50
Duodenal diverticulum, *n* (%)	8 (32.0)	9 (22.0)	0.40
Indication for EST, *n* (%)			0.022
Bile duct stone(s)	14 (56.0)	18 (43.9)	
Biliary drainage (malignancy/cholangitis)	4 (16.0)	19 (46.3)	
Other (cholangioscopy, ampullary lesion, etc.)	7 (28.0)	4 (9.8)	
Antithrombotic therapy, *n*			0.78
Any antithrombotic agent	7	10	
Aspirin/cilostazol	4	6	
Warfarin	2	0	
DOAC	1	3	
Thienopyridine	0	1	
International normalized ratio, median (IQR)	1.17(1.08–1.33)	1.15(1.08–1.23)	0.47
Platelet count, ×10 ^4^ /μL, median (IQR)	23.2(17.5–31.6)	23.4(19.7–30.9)	0.62
Hemoglobin, g/dL, median (IQR)	12.2(10.7–13.1)	12.2(11.1–13.6)	0.50
Glasgow–Blatchford score, median (IQR)	1(1–5)	1 (0–3)	0.28
AST, U/L, median (IQR)	39.0(17.0–132.3)	67.0(28.0–146.0)	0.17
ALT, U/L, median (IQR)	32.5(14.0–119.8)	64.0(18.0–180.0)	0.16
Amylase, U/L, median (IQR)	62.5(50.8–100.0)	80.0(44.0–116.0)	0.79

**Table 2:** Clinical outcomes.

Outcome	Hemospray ( *n* = 25)	Balloon ( *n* = 41)	*P* -value
Management of antithrombotic therapy (among users), *n* Interrupted/Heparin bridging/Uninterrupted	4/0/3	6/0/4	0.96
EST incision size, *n* (%)			0.79
Small	16 (64.0)	24 (58.5)	
Medium	9 (36.0)	16 (39.0)	
Large	0	1 (2.4)	
Initial hemostasis, *n* (%, 95% CI)	24/25 (96.0)	30/41 (73.2)	0.023
	95% CI 80.5–99.3	95% CI 58.1–84.3	
Risk difference (Hemospray − Balloon), %	+22.8 (95% CI 4.3–41.4)	—	
Adjusted OR for initial hemostasis (Firth)†	7.7 (95% CI 1.5–84.2)	—	0.013
Propensity-matched analysis (20 pairs)	19/20 (95.0)	16/20 (80.0)	0.375
Rescue device used, *n*			
Self-assembling peptide (PuraStat)	0	7	
Thermal coagulation	0	3	
SEMS deployment	0	1	
Balloon tamponade	1	0	
Overall endoscopic hemostasis, *n* (%)	25 (100)	41 (100)	-
Adverse events within 7 days, *n* (%)			
Any adverse event	3 (12.0)	9 (22.0) 8 (19.5)	0.36
Acute pancreatitis	2 (8.0)	1 (2.4)	0.30
Acute cholangitis	1 (4.0)		>0.99
Rebleeding within 30 days, *n* (%)	3 (12.0; 95% CI 4.2–30.0)	1 (2.4; 95% CI 0.4–12.6)	0.14
Hemostasis for rebleeding			
Clip	2	0	
Hemospray	2	0	
Self-assembling peptide	1	0	
Thermal coagulation + SEMS	0	1	

Initial hemostasis was achieved in 24 of 25 patients (96.0%, 95% CI 80.5–99.3) in the Hemospray group versus 30 of 41 patients (73.2%, 95% CI 58.1–84.3) in the balloon group (risk difference +22.8%, 95% CI 4.3–41.4; *P* = 0.023). Firth-penalized multivariable logistic regression adjusted for age, antithrombotic therapy, EST incision size, EST indication, and Glasgow–Blatchford score supported the direction of effect (adjusted odds ratio 7.7, 95% CI 1.5–84.2; *P* = 0.013). The wide upper confidence limit reflects quasi-separation with only one primary-outcome failure in the Hemospray group; the regression therefore should be interpreted as supportive of the direction of the effect rather than as a precise estimate of effect size. In the prespecified 1:1 propensity-score–matched supportive sensitivity analysis (caliper 0.109; 20 pairs formed), initial hemostasis remained directionally higher with Hemospray (19/20 [95.0%] vs. 16/20 [80.0%]; *P* = 0.375). Among patients with failed initial hemostasis, bleeding was successfully controlled with balloon tamponade ( *n* = 1) in the Hemospray group and with self-assembling peptides ( *n* = 7), thermal coagulation ( *n* = 3), and SEMS deployment ( *n* = 1) in the balloon group. Accordingly, overall endoscopic hemostasis was achieved in all 66 patients in both groups (100% vs. 100%); the group-level advantage in this cohort was therefore confined to initial hemostasis and avoidance of rescue-device use, not to overall endoscopic hemostasis.

### Safety and Rebleeding

Adverse events within seven days occurred in 3 of 25 patients (12.0%, 95% CI 4.2–30.0) in the Hemospray group and in 9 of 41 patients (22.0%, 95% CI 12.0–36.7) in the balloon group ( *P* = 0.36). Acute pancreatitis developed in 2 (8.0%) and 8 (19.5%) patients, respectively ( *P* = 0.30); acute cholangitis in 1 (4.0%) and 1 (2.4%) patient, respectively ( *P* > 0.99). Post-procedural imaging (CT) was obtained only when a pancreatobiliary adverse event was clinically suspected. Two Hemospray-group patients underwent CT (one with acute pancreatitis, one with acute cholangitis); in both, no new biliary or pancreatic duct dilation was observed compared with preprocedural imaging. Our safety data therefore permit only the narrower statement that, in the evaluated cases, no clinically apparent pancreatobiliary ductal obstruction was detected; asymptomatic, transient ductal obstruction cannot be excluded and would require protocol-mandated post-procedural imaging to address.

All rebleeding events occurred within 24 h after the index procedure. The cumulative rebleeding rates at 24, 72 h, and 30 days were identical: 3 of 25 patients (12.0%) in the Hemospray group and 1 of 41 patients (2.4%) in the balloon-tamponade group ( *P* = 0.14). No additional rebleeding occurred between 24 h and 30 days, and all rebleeding events were successfully managed endoscopically.

### Exploratory Device-cost Comparison

Mean device expenditure, including the first-line and rescue hemostatic devices, was US$314.2 ± 63.0 in the Hemospray group and US$414.7 ± 277.5 in the balloon-tamponade group. The mean difference was −US$100.4 (95% CI −188.9 to −12.0; *P* = 0.031). This exploratory comparison was restricted to device costs and should not be interpreted as a formal cost-effectiveness analysis ( Table 3 ).

**Table 3:** Exploratory comparison of hemostatic device expenditure.

Outcome	Hemospray ( *n* = 25)	Balloon ( *n* = 41)	Effect estimate	*P* -value
Mean device-related cost (initial + rescue), US$ ± SD	314.2 ± 63.0	414.7 ± 277.5	Mean difference −100.4 (95% CI −188.9 to −12.0)	0.031
Median cost, US$ (IQR)	301.7 (301.7–301.7)	314.9 (314.9–314.9)	–	–

## Discussion

To our knowledge, this is one of the earliest studies to specifically evaluate the safety and effectiveness of topical hemostatic powder applied to the ampulla of Vater in the setting of immediate post-EST bleeding. Using a standardized papillary application protocol, Hemospray was associated with a higher rate of initial hemostasis and a lower requirement for rescue devices than balloon tamponade. These findings persisted after Firth-penalized multivariable adjustment for baseline covariates, including EST indication, and were directionally consistent in a prespecified propensity-score–matched supportive sensitivity analysis. However, overall endoscopic hemostasis was achieved in 100% of patients in both groups, so the advantage of Hemospray in this cohort is specifically for initial control and avoidance of rescue-device use rather than for overall hemostasis. Given the wide confidence interval of the Firth estimate and the limited statistical power of the 20-pair propensity-score–matched analysis, these adjusted analyses should be interpreted only as supporting the direction of the observed association rather than as providing confirmatory evidence or a precise estimate of effect size. Moreover, propensity-score matching cannot address temporal changes or unmeasured confounders inherent in a historical-control design. However, because treatment allocation was determined entirely by the historical period, the observed association cannot be attributed solely to the hemostatic modality. Changes in referral patterns, case mix, operator experience, and other unmeasured temporal factors may have contributed to the difference in initial hemostasis. The small sample size, particularly the 25 patients in the Hemospray group, limited the precision of the effect estimates. Therefore, the observed association should be regarded as exploratory and hypothesis-generating rather than as definitive evidence of comparative effectiveness.

Several endoscopic techniques can be employed to treat immediate post-EST bleeding, including thermal coagulation, balloon tamponade, SEMS deployment, and hemoclipping. Thermal coagulation may cause additional trauma to the ampulla of Vater, potentially increasing the risks of cholangitis and pancreatitis. 7 Hemoclipping has become more feasible and widely applicable with recent advances in device technology. 8 In a recent series of 41 patients with urgent ( *n* = 21) or delayed bleeding ( *n* = 20) after EST, a novel duodenoscope-deliverable clip (SureClips; Micro-Tech, Nanjing, China) achieved complete hemostasis in 91.7% of cases, with no reported perforation, pancreatitis, or inadvertent biliopancreatic clipping. 8 Nevertheless, clipping at the papilla remains demanding for nonexpert endoscopists because of the limited duodenal working space and obscured endoscopic view during active bleeding. SEMS deployment, by contrast, is straightforward once biliary access has been secured. Inoue et al. 9 reported a primary hemostasis rate of 100% and a rebleeding rate of 5% with early SEMS placement for massive post-EST bleeding, compared with 31% rebleeding with conventional combination therapy ( *P* = 0.023), together with shorter procedure times and reduced transfusion requirements.

SEMS placement, however, entails higher procedural costs and typically requires a second endoscopic session for stent removal. More importantly, stent-related adverse events, particularly post-ERCP pancreatitis, remain a clinical concern because the stent is deployed across the papilla, potentially compressing the pancreatic duct orifice. This risk may be accentuated in the context of post-EST bleeding, in which sphincterotomy is sometimes incomplete because of poor visualization or hemodynamic instability; residual sphincter muscle or papillary edema may then increase the likelihood of pancreatic duct compression after stent placement. Therefore, less invasive techniques with favorable safety profiles remain desirable in routine practice.

Topical hemostatic agents are comparatively easy to deploy within the duodenum. PuraStat has been reported for this indication 12 ; although high success rates have been described, the gel may dislodge and migrate into the third part of the duodenum, and specific technical nuances are required to prevent dislocation. 13 In the present study we positioned Hemospray as an alternative to PuraStat rather than as its replacement: for patients in whom the papillary orientation is unfavorable for gel retention, or when local conditions make sustained gel coverage difficult, powder spraying in a sweeping, tangential pattern offers wider coverage with a lower technical threshold. Indeed, one Hemospray-group patient in whom initial powder application failed was successfully rescued with balloon tamponade, and PuraStat remained effective as a rescue modality in the balloon group. A recent meta-analysis of five randomized controlled trials including 362 patients reported no significant difference in primary hemostasis between Hemospray and standard techniques ( *P* = 0.2) but a lower rate of hemostatic failure with Hemospray ( *P* = 0.01). 17 Taken together, these data support the notion that Hemospray is a clinically useful, complementary option for gastrointestinal hemostasis.

An important practical caveat is that Hemospray is difficult to deliver once the delivery catheter becomes moist. Within the narrow duodenal lumen, specific technical modifications, such as those implemented in the present protocol, are required to ensure effective delivery. In our cohort, these modifications allowed successful delivery in every case without premature catheter occlusion.

A second concern is that the powder is dispersed not only onto the bleeding point but also across the entire papillary region, raising theoretical questions about obstruction of the biliary and, especially, pancreatic duct orifices. Because post-procedural imaging was obtained only when pancreatobiliary adverse events were clinically suspected, our results can support only the narrower statement that, in the evaluated cases, no new biliary or pancreatic ductal dilation was observed; asymptomatic, transient ductal obstruction cannot be excluded. Definitive safety conclusions about papillary Hemospray therefore require prospective studies with protocol-mandated post-procedural CT or magnetic resonance cholangiopancreatography, and the present data should not be cited as proof that papillary Hemospray is uniformly safe with respect to ductal obstruction. A plausible mechanism that might nevertheless favor safety is that any transient coverage of the orifice could be washed out by antegrade biliary and pancreatic juice flow, whereas at the bleeding surface the powder adheres to the injured mucosa and promotes platelet aggregation; this, however, remains a hypothesis that requires direct imaging confirmation.

All rebleeding events occurred within 24 h after the index procedure, and no additional rebleeding occurred through 30 days. Accordingly, the cumulative rebleeding rates at 24, 72 h, and 30 days were 12.0% in the Hemospray group and 2.4% in the balloon-tamponade group. Although the absolute number of events was small and the between-group difference was not statistically significant, the numerically higher early rebleeding rate after Hemospray raises a clinically relevant concern regarding the durability of topical powder hemostasis. Hemospray forms a cohesive barrier that promotes immediate clot formation, but local dilution of clotting factors and clot dislodgement have been described and may limit durability. 16 17 This is consistent with our observation that the group-level benefit of Hemospray in this cohort is concentrated at the time of initial control rather than at 30-day durability. The advantage of Hemospray is therefore best framed as improved initial hemostasis and reduced rescue-device use during the index procedure, rather than as improved medium-term or durable hemostasis; this framing aligns the clinical message with what the data can support. Most bleeding events in this cohort were classified as mild according to the ASGE lexicon. However, this classification is based primarily on subsequent clinical consequences, including transfusion, intensive care, angiographic intervention, and duration of hospitalization, and should not be equated with transient minor oozing at the time of EST. In the present study, self-limited oozing that resolved without hemostatic intervention was excluded, and treatment was restricted to oozing that persisted despite irrigation, suction, and 3 min of observation. Nevertheless, because the intensity of oozing and the exact observation duration were not systematically documented in all retrospective records, some episodes of persistent oozing might have ceased spontaneously with a longer observation period. The clinical benefit of Hemospray may therefore be limited in patients with low-intensity bleeding. Furthermore, because overall endoscopic hemostasis was achieved in all patients in both groups, the observed advantage should be interpreted as improved initial control and reduced rescue-device use during the index procedure rather than as improved definitive hemostasis. Whether these findings apply to moderate or severe post-EST bleeding remains uncertain. The exploratory device-cost comparison showed lower mean device expenditure with the Hemospray-first strategy. However, only the costs of the first-line and rescue hemostatic devices were included. Hospitalization, transfusion, readmission, personnel, procedural, and indirect costs were not evaluated. These findings therefore do not establish the overall cost-effectiveness of Hemospray.

Our study has several important limitations. First, this was a single-center, retrospective historical-control study in which the treatment groups were defined by different time periods, with a 15-month balloon-tamponade period and a 4-month Hemospray period. The higher monthly ERCP and EST volumes during the later period followed the establishment and dissemination of a structured referral pathway, which may have altered referral patterns and case mix. Therefore, selection bias and temporal bias related to changes in patient referral, disease characteristics, periprocedural management, operator experience, and secular trends in ERCP practice cannot be fully excluded. Although all included cases were documented as persistent nonpulsatile oozing, the intensity of oozing and strict adherence to the 3-minute pretreatment observation period were not systematically recorded as research variables and could not be independently verified in every case. Similarly, although approximately 3 min of post-treatment observation represented the institutional standard for confirming initial hemostasis, the exact observation duration was explicitly documented in only a subset of cases and was not systematically time-stamped. Initial hemostasis was therefore retrospectively adjudicated based on documentation of bleeding cessation and the absence of immediate rescue therapy, introducing a potential risk of outcome misclassification. Firth-penalized regression and propensity-score matching may reduce confounding attributable to the measured baseline variables included in the models, but they cannot eliminate residual or unmeasured confounding inherent in a historical-control comparison. In addition, because the cohort predominantly comprised cases classified as mild according to the ASGE lexicon, the clinical impact and generalizability of the findings may be limited. Although episodes of self-limited oozing were excluded, some treated episodes of persistent oozing might have ceased spontaneously with a longer observation period. The present results therefore cannot establish that immediate hemostatic intervention is necessary for all mild post-EST bleeding or that Hemospray is effective in moderate or severe bleeding. In addition, most procedures involved small or medium ESTs, and no patient in the Hemospray group underwent large EST. Therefore, the present findings should not be extrapolated to bleeding after large EST, and the efficacy and safety of Hemospray in this setting remain uncertain. Although the operator eligibility and expert-supervision structure remained unchanged throughout both study periods, the individual operators were not necessarily identical and may have accumulated additional experience over time. Operator-related temporal effects therefore cannot be completely excluded. Second, the sample size was small, particularly in the Hemospray group, and no formal sample-size calculation was performed. This resulted in wide confidence intervals, quasi-separation in the Firth model, and only 20 propensity-score–matched pairs. The adjusted and matched analyses were therefore underpowered and should be interpreted as supportive, exploratory analyses rather than confirmatory comparisons. Finally, CT was performed only when clinically indicated, and the safety statement about pancreatobiliary duct obstruction is therefore restricted to evaluated cases; asymptomatic or transient obstruction cannot be excluded, and prospective studies with protocol-mandated post-procedural imaging are required.

In conclusion, among patients with persistent nonpulsatile post-EST oozing that did not resolve during a 3-minute observation period, a standardized Hemospray-first strategy was associated with more frequent initial bleeding control and reduced rescue-device use compared with balloon tamponade. However, overall endoscopic hemostasis was achieved in all patients in both groups, and early rebleeding within 24 h was numerically more frequent after Hemospray, with no additional events through 30 days. These findings therefore do not establish the superiority of Hemospray for definitive or durable hemostasis. Prospective multicenter studies with standardized endpoint assessment and longer follow-up are required.
